# Establishment of a simplified *in vitro* porcine blood–brain barrier model with high transendothelial electrical resistance

**DOI:** 10.1016/j.brainres.2012.06.057

**Published:** 2013-07-12

**Authors:** Adjanie Patabendige, Robert A. Skinner, N. Joan Abbott

**Affiliations:** a King's College London, Institute of Pharmaceutical Science, BBB Group, Franklin Wilkins Building, 150 Stamford St, London SE1 9NH, UK; b Faculty of Life Sciences, University of Manchester, Manchester, UK

**Keywords:** ACM, astrocyte-conditioned medium, ALP, alkaline phosphatase, AMT, adsorptive-mediated trancytosis, BCA, bicinchoninic acid, bFGF, basic fibroblast growth factor, BPDS, bovine plasma derived serum, GAPDH, glyceraldehyde-3-phosphate dehydrogenase, GLUT-1, glucose transporter-1, NGS, normal goat serum, PBEC, porcine brain endothelial cells, pCPT-cAMP, 8-(4-parachlorophenylthio)-cAMP, P-gp, P-glycoprotein, PKA, protein kinase A, *p*NPP, *p*-nitrophenyl phosphate, QC, quality control, RO-20-1724, 4-(3-butoxy-4-methoxybenzyl)-2-imidazolidinone, RMT, receptor-mediated trancytosis, TEER, transendothelial electrical resistance, Blood–brain barrier, Brain endothelium, Transendothelial electrical resistance, Tight junction, Transport, Permeability

## Abstract

Good *in vitro* blood–brain barrier (BBB) models that mimic the *in vivo* BBB phenotype are essential for studies on BBB functionality and for initial screening in drug discovery programmes, as many potential therapeutic drug candidates have poor BBB permeation. Difficulties associated with the availability of human brain tissue, coupled with the time and cost associated with using animals for this kind of research have led to the development of non-human cell culture models. However, most BBB models display a low transendothelial electrical resistance (TEER), which is a measure of the tightness of the BBB. To address these issues we have established and optimised a robust, simple to use *in vitro* BBB model using porcine brain endothelial cells (PBECs). The PBEC model gives high TEER without the need for co-culture with astrocytes (up to 1300 Ω cm^2^ with a mean TEER of ∼800 Ω cm^2^) with well organised tight junctions as shown by immunostaining for occludin and claudin-5. Functional assays confirmed the presence of high levels of alkaline phosphatase (ALP), and presence of the efflux transporter, P-glycoprotein (P-gp, ABCB1). Presence of the breast cancer resistance protein (BCRP, ABCG2) was confirmed by TaqMan real-time RT-PCR assay. Real-time RT-PCR assays for BCRP, occludin and claudin-5 demonstrated no significant differences between batches of PBECs, and also between primary and passage 1 PBECs. A permeability screen of 10 compounds demonstrated the usefulness of the model as a tool for drug permeability studies. Qualitative and quantitative results from this study confirm that this *in vitro* porcine BBB model is reliable and robust; it is also simpler to generate than most other BBB models.

*This article is part of a Special Issue entitled Electrical Synapses.*

## Highlights

► We have established and tested an *in vitro* porcine blood–brain barrier (BBB) model. ► The model demonstrates high transendothelial electrical resistance and other key BBB features. ► It is suitable as a blood–brain barrier drug permeability screening tool.

## 1 Introduction

The blood–brain barrier is formed by brain endothelial cells lining cerebral microvessels, and performs a combination of physical, transport and enzymatic barrier functions (Abbott et al., 2010). The physical barrier is largely the result of extremely tight *zonulae occludentes* (tight junctions), which seriously restrict the paracellular flux of small hydrophilic molecules (Tsukita et al., 2001; Wolburg and Lippoldt, 2002). The transport barrier results from a combination of specific membrane carrier systems for uptake and efflux that regulate small molecular traffic at apical (luminal) and basal (abluminal) membranes (Begley, 2004; Hawkins et al., 2002; Hawkins et al., 2006), together with receptor-mediated and absorptive-mediated transcytosis (RMT, AMT) that mediate the transfer of small amounts of larger molecules such as peptides and proteins (Hervé et al., 2008; Wolburg et al., 2009). The enzymatic barrier results from the presence on and within brain endothelial, of ecto- and endo-enzymes capable of metabolising endogenous and exogenous compounds (Abbott et al., 2006; Persidsky et al., 2006). The net result of all three barrier functions is protection of the brain from potentially toxic or neuroactive agents capable of disturbing neural function, and a contribution to homoeostatic regulation of the brain microenvironment that is essential for neural activity and integration.

Increased understanding of BBB function has come from careful study *in vivo*, traditionally using animal models, and increasingly involving minimally invasive investigation, where the technologies allow, in human subjects (Hawkins and Egleton, 2007; Rebeles et al., 2006). However, for elucidating the detailed cellular and molecular mechanisms involved, *in vitro* models play an important part. A number of cell culture models of the BBB have been developed from a variety of species (de Boer and Gaillard, 2002; Deli et al., 2005; Garberg et al., 2005; Gumbleton and Audus, 2001; Reichel et al., 2003). Although the aim in many cases is to understand the human condition, for the present, human brain endothelial models of sufficient yield, tightness and reproducibility have not been available. Several immortalised human BBB models have been developed with good expression of BBB markers but generally have a lower transendothelial electrical resistance (TEER) than most animal models (Förster et al., 2008; Grab et al., 2004; Sano et al., 2010; Weksler et al., 2005). Models derived from rat provide useful comparison with *in vivo* studies, the rat still being the most widely used animal model for experimental study, including for pharmaceutical applications and pharmacokinetic investigation (Abbott et al., 1992; Perrière et al., 2005; Perrière et al., 2007; Roux and Couraud, 2005). Mouse models are opening up the field for applications using genetically modified animals (Förster et al., 2005; Omidi et al., 2003; Shayan et al., 2011). However, models from rat and mouse are labour-intensive and low yield, so that for higher yield applications including medium-throughput screening studies, bovine and porcine brain endothelium have been the models of choice (Bowman et al., 1983; Cecchelli et al., 1999; Franke et al., 2000; Gaillard et al., 2001; Miller et al., 1992; Smith et al., 2007; Zhang et al., 2006).

We recently adopted a porcine brain endothelial cell (PBEC) model first developed at Eisai Laboratories (London) by Dr. Louise Morgan and colleagues, based on a successful earlier bovine brain endothelial cell model (Rubin et al., 1991). A feature of this method of cell preparation is the two-stage filtration using nylon meshes that catch the microvessels, followed by a subculturing step that improves purity. In the earlier development of the method, optimal BBB phenotype and barrier tightness were achieved by growth in supplemented medium, including astrocyte-conditioned medium. We have made further modifications to the method, making it significantly simpler to prepare (by avoiding the use of astrocytes or astrocyte-conditioned medium) and by eliminating contaminating cells such as pericytes. Here we describe important features of the model, especially high TEER and retention of other key BBB features, and outline applications including use as a tool for drug screening. A report on use of a variant of the model to examine receptor-mediated transport of interleukin-1β has been published (Skinner et al., 2009).

## 2 Results

### 2.1 Cell growth and morphology

**Fig. 1:**
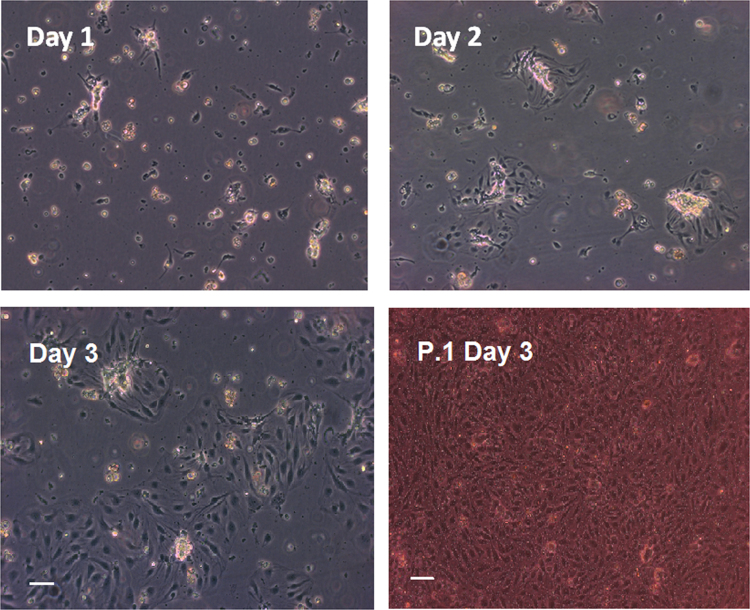
Phase contrast images of a primary porcine brain endothelial cell (PBEC) culture. The cells were treated with 4 μg/ml puromycin for three days to remove contaminating cells (as described in Section 4.3). Porcine brain endothelial cells start to migrate from microvessel fragments from day 1. By day 3, the culture is about 70% confluent and can be passaged at this stage. Bottom right image shows confluent P.1 PBEC cultures on Transwell inserts, three days after passaging (six days from thawing). Scale bar: 50 μm.

Isolated porcine brain endothelial microvessel fragments attached to culture flasks coated with collagen/fibronectin within a few hours of plating. PBECs started growing out of these microvessel fragments in clusters and became ∼70–80% confluent after three days in culture (Fig. 1). Contaminating cells such as pericytes and astrocytes were not observed in the porcine brain endothelial cell monolayers under phase-contrast microscope following the use of puromycin to purify the cultures. Confluent monocultures of porcine brain endothelial cells have an elongated cobblestone-shaped morphology, although not generally so clearly spindle-shaped as reported for rat and bovine brain endothelial cell cultures.

### 2.2 Tight junction proteins and paracellular barrier

**Fig. 2:**
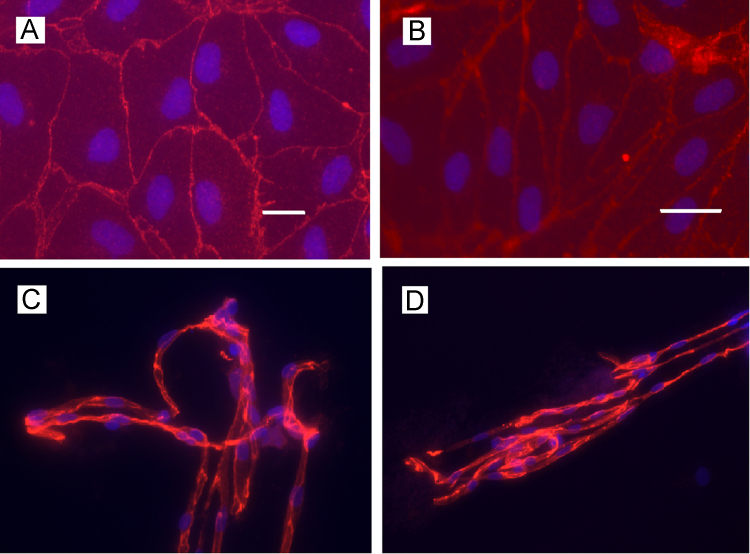
Fluorescence micrograph of the immunocytochemical localisation of occludin and claudin-5 in P.1 PBEC and porcine brain microvessels. P.1 PBEC were grown on glass cover slips (A, B) then stained for tight-junction proteins occludin (A, scale bar: 50 μm) and claudin-5 (B, scale bar: 20 μm). Porcine brain microvessels were isolated from fresh porcine brain tissue onto glass coverslips using the ‘tissue print’ method (Section 4.5). (C) Occludin (viewed at 40×magnification); (D) claudin-5 (20×magnification); nuclei counterstained with Hoescht 33258.

Cultures of passage 1 (P.1) PBECs formed confluent monolayers of non-overlapping contact-inhibited cells. Immunocytochemical studies revealed clear marginal staining for occludin and claudin-5 (Fig. 2 A and B respectively) consistent with well-organised tight junctions, characteristic of the BBB. Clear staining for occludin and claudin-5 (Fig. 2, C and D respectively) was also seen in freshly isolated porcine brain microvessels.

**Fig. 3:**
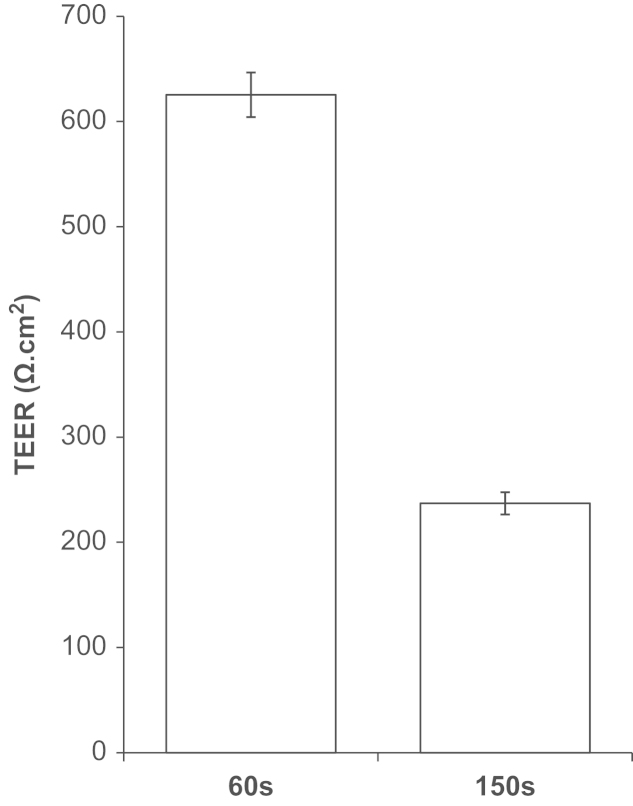
TEER differences between 60s and 150s fractions from the same batch of PBEC. Puromycin-treated PBEC were passaged and grown on 12 mm diameter Transwell Clear filter inserts (0.4 μm pore size) for three days. Cells were treated with supplements (CPT-cAMP, RO-20-1724 and hydrocortisone) for 24 h and TEER measured. TEER of a ‘blank’ cell-free insert has been subtracted from all values. Mean±SEM (*n*=6).

P.1 PBECs from the ‘60s’ fraction gave higher TEER than the ‘150s’ fraction (Fig. 3) and were used for all experiments described here. P.1 PBECs (60s) cultured on Transwell Clear inserts were found to give a maximum TEER of ∼1300 Ω cm^2^ (mean=789±18 Ω cm^2^, *n*=91 inserts in 24 independent experiments and a minimum apparent permeability (*P*_app_) to [^14^C]sucrose of 3.0×10^−6^ cm/s (mean=6.07±0.32×10^−6^ cm/s, *n*=29 inserts in four independent experiments). The quality control (QC) benchmark for permeability was set at a TEER of 500 Ω cm^2^ and a *P*_app_ sucrose of 8×10^−6^ cm/s. P.1 PBECs always achieved these targets when the strict preparative methodology was followed, including following the QC benchmarks for morphology and confluence level (Table 1).

**Fig. 4:**
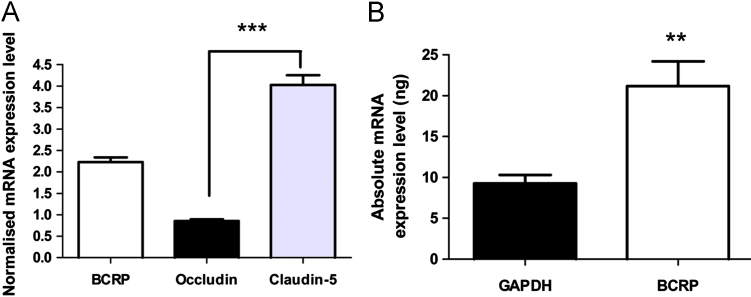
mRNA expression of breast cancer-resistance protein (BCRP), occludin and claudin-5 in P.1 PBEC. (A) Normalised mRNA expression levels of BCRP, occludin and claudin-5 for P.1 PBEC cultures. P.1 PBEC mRNA data for each gene were normalised against GAPDH (mean±SEM, *n*=12; independent-sample *t*-test; ^***^p<0.0001). Statistical significance between the three genes was determined by one-way ANOVA, followed by Dunnet's test for equal variances (^***^p<0.0001). (B) Absolute mRNA expression levels of GAPDH and BCRP. mRNA transcripts are from 12 samples (mean±SEM; independent-sample *t*-test; ^**^p<0.01).

TaqMan real-time RT-PCR analysis confirmed the expression of occludin and claudin-5 in P.1 PBECs. When normalised against GAPDH, mRNA expression level was significantly higher for claudin-5 than for occludin (Fig. 4A).

### 2.3 BBB transporters and alkaline phosphatase

#### 2.3.1 P-glycoprotein

**Fig. 5:**
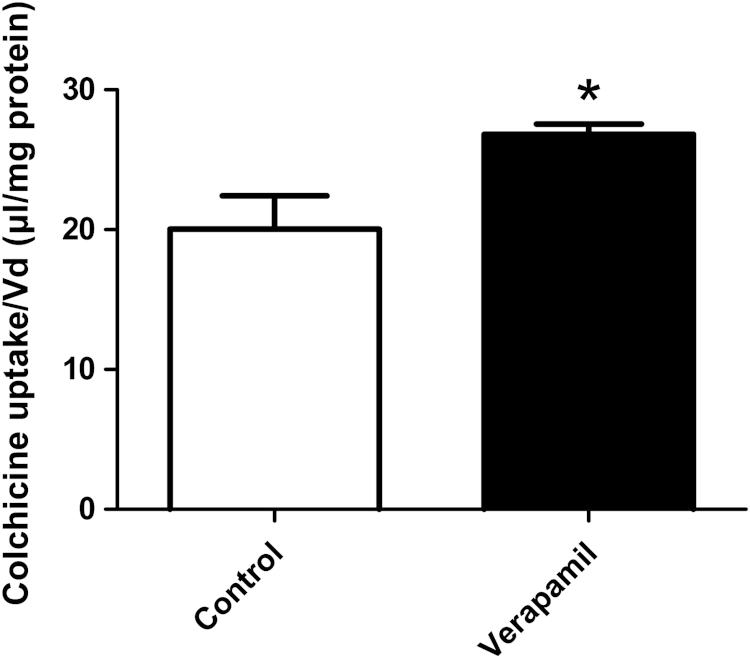
Assessment of P-glycoprotein function in P.1 PBEC. [^3^H]Colchicine uptake assay for P-gp activity. Mean±SEM (*n*=6). Independent-sample *t*-test; ^*^p<0.05 compared to the control. Colchicine uptake (*V*_d_, volume of distribution) in presence of P-gp inhibitor verapamil showed a ‘factor increase’ (*V*_d_ in presence of verapamil/*V*_d_ in control) of 1.34 compared to the control without inhibitor, evidence for presence of functional P-gp.

P-glycoprotein (P-gp, ABCB1) is an efflux transporter located on the luminal membrane of the endothelial cells of the BBB. Uptake of [^3^H]colchicine, a P-gp substrate, into confluent P.1 PBECs is shown in Fig. 5. Addition of 50 μM P-gp inhibitor verapamil to the incubation medium caused a significant increase (*p*<0.05) in colchicine uptake into P.1 PBECs compared to control cells, evidence for presence of functional P-gp.

#### 2.3.2 Breast cancer-resistance protein

TaqMan real-time RT-PCR assays confirmed the presence of the efflux transporter breast cancer-resistance protein (BCRP) in P.1 PBECs. Normalisation against GAPDH mRNA expression levels in P.1 PBECs showed that BCRP expression is significantly higher than occludin (*p*<0.0001) and lower than claudin-5 (*p*<0.0001; Fig. 4A). The mRNA transcript level of BCRP in P.1 PBECs was twice that of GAPDH (*p*<0.01; Fig. 4B).

#### 2.3.3 Alkaline phosphatase

**Fig. 6:**
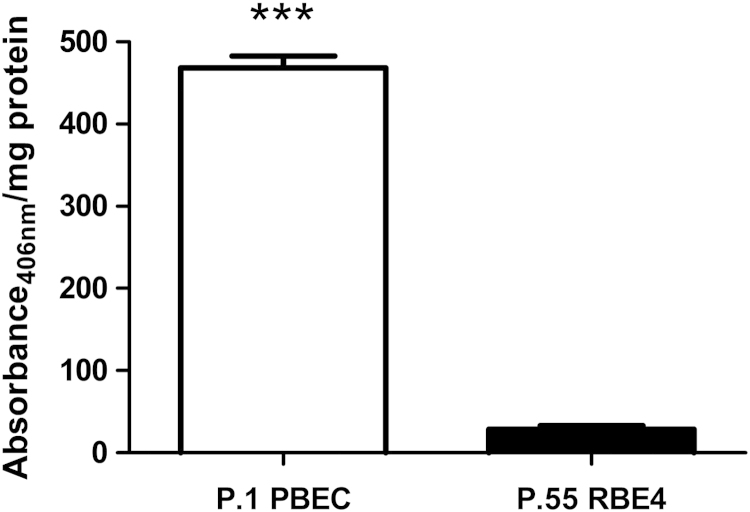
Comparison of ALP activity between P.1 PBEC and P.55 RBE4 cells. ALP assay was performed on confluent cells using *p*NPP as ALP substrate as described in Section 4.10. ALP activity of P.1 PBEC was over 20 times greater than in P.55 RBE4 cells (mean±SEM, *n*=24; independent-sample *t*-test; ^***^p<0.0001).

Alkaline phosphatase (ALP) activity of P.1 PBECs was measured using *p*-nitrophenyl phosphate (*p*NPP) as substrate. Significantly higher levels (*p*<0.0001) of ALP activity were detected in P.1 PBECs than in an immortalised rat brain endothelial cell line, RBE4 (Fig. 6).

### 2.4 Maintenance of BBB features after passaging; mRNA data

**Fig. 7:**
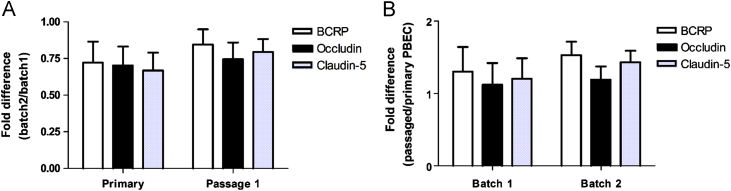
Comparison of relative mRNA expression levels of BCRP, occludin and claudin-5 in PBEC cultures. (A) Relative mRNA expression levels between batches of PBEC. The results are expressed as ‘fold difference’ ratio between batch 2 and batch 1 PBEC cultures (mean±SEM, *n*=6). (B) Relative mRNA expression levels between primary and P.1 PBEC cultures within each batch. Results are expressed as ‘fold difference’ ratio between passage 1 and primary PBEC cultures (mean±SEM, *n*=6). Statistical significance was determined by two-way ANOVA and showed no significant differences between batches or cultures (primary *vs.* passage 1) of PBECs.

Normalised mRNA data were used to calculate ‘fold differences’ to monitor batch to batch differences. The results showed no significant differences in mRNA expression levels of BCRP, occludin and claudin-5 between batches of PBEC cultures (based on 2-fold difference threshold; Fig. 7A) for all genes assayed. Batch2/batch1 fold difference ratio was less than 2-fold, which confirms the stability of the expression levels of the genes between batches. Passage 1/primary fold difference ratio was calculated to assess differences in mRNA expression levels in PBECs in different batches. The results showed no significant differences in mRNA expression levels between primary and P.1 PBEC cultures for either batch 1 or 2 (Fig. 7B) for all genes assayed. Mean P.1/primary fold difference ratio was less than 1.6, below the 2-fold difference of mRNA expression considered significant.

### 2.5 Permeability screening of compounds

**Fig. 8:**
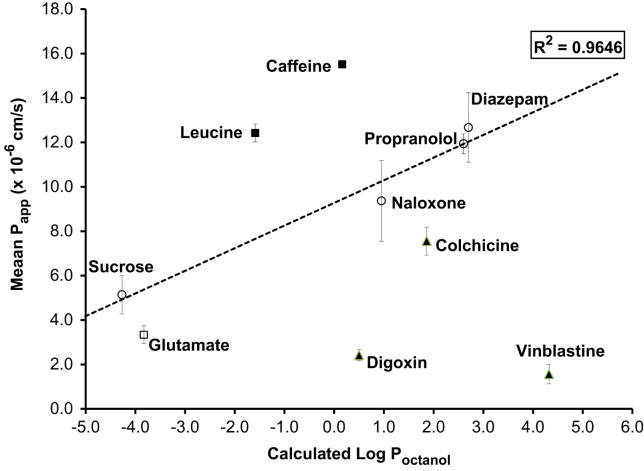
Correlation between *in vitro P*_app_ (apical to basal direction) for P.1 PBEC and calculated Log *P*_octanol_. P.1 PBEC were grown on Transwell Clear inserts and were used after treatment with cAMP, RO-20-1724 and hydrocortisone for 24 h (*n*=3 experiments, 9–12 inserts for each compound). Permeability assays were performed as described in Section 4.8 and 4.15. Calculated Log *P*_octanol_ was obtained from http://www.syrres.com/eSc/est_kowdemo.htm. Solute permeation: open circles, passive; closed squares, uptake; closed triangles, ABC-mediated efflux; open square, glutamate – subject to both uptake and efflux, see Section 2.5.

The plot of *P*_app_ *vs*. calculated Log *P*_octanol_ (Fig. 8) showed that compounds predicted to move by passive permeation either paracellularly or transcellularly (sucrose, naloxone, propranolol, diazepam) had *P*_app_ that was a linear function of calculated Log *P*_octanol_, with *R*^2^=0.96. Leucine, taken up by LAT-1 (large neutral amino acid carrier), and caffeine (saturable carrier-mediated transport mechanism) (McCall et al., 1982) are both clear outliers above the line as predicted (permeation >predicted from Log *P*), while the four compounds that are known substrates for either ATP-dependent efflux transporters (digoxin, colchicine and vinblastine for P-gp) or basolateral Na-dependent secondary active transport (glutamate, substrate for excitatory amino acid transporter, EAAT) are clear outliers below the line as predicted (permeation<predicted from Log *P*).

## 3 Discussion

### 3.1 BBB phenotype of PBEC model

We have optimised and characterised an *in vitro* BBB model using PBECs that fits the requirements of a generally applicable *in vitro* BBB model (Garberg et al., 2005; Prieto et al., 2004). PBECs are readily available (with a yield of ∼1×10^7^ cells per brain) and are easy to culture. Addition of puromycin to eliminate contaminating cells in the culture was one of the most significant steps in the method, with other improvements such as careful dissection of brains to remove meninges and white matter. This led to a significant increase in TEER because of the purity of cultures. Growth on rat-tail collagen/fibronectin gave good adhesion and improved uniform growth to confluence. The method generated PBECs with high TEER (up to 1300 Ω cm^2^, with an average of ∼800 Ω cm^2^) which can be used reliably in experiments. The minimum apparent permeability of the confluent cell monolayers to [^14^C]sucrose was 3×10^−6^ cm/s, with permeability below 8×10^−6^ cm/s among all porcine cultures tested. The mean TEER in our PBEC cultures is similar to that obtained by Franke et al., 2000 and ∼3–10 times higher than reported for other porcine models when PBECs are cultured without astrocytes or ACM (*e.g.* Jeliazkova-Mecheva and Bobilya, 2003; Smith et al., 2007; Zhang et al., 2006). Even when cultured with astrocytes or ACM, some of these porcine models are unable to achieve TEER comparable to our model. Furthermore, permeability to [^14^C]sucrose in the present model is lower than or comparable to that of other porcine models. Immunochemical studies revealed clear marginal staining for occludin and claudin-5 in P.1 PBECs and edge staining in porcine brain microvessels, consistent with well-organised tight junctions. Functional assays confirmed the presence of the brain efflux transporter, P-gp and high levels of ALP activity in P.1 PBECs. Functional ALP has been reported in RBE4 cells (Roux et al., 1994) and was increased when astrocyte-conditioned medium (ACM) and retinoic acid were used (el Hafny et al., 1996). In our model, ALP activity in P.1 PBECs without any astrocytic factors was over 20 times greater than in P.55 RBE4 cells. We chose RBE4 cells for comparison as this cell line is widely used and well characterised, and has been used in previous studies of ALP. The loss of activity in BBB enzymes such as ALP and gamma glutamyl transpeptidate in primary PBECs is well documented (Beuckmann et al., 1995; Meyer et al., 1990, 1991). Therefore, the comparison here demonstrates the quality of the present model, retaining ALP activity in culture even after seven days from isolation. Raising intracellular cAMP in P.1 PBECs could have contributed to the high level of ALP activity in our model, as it has been shown that elevating cAMP can induce ALP activity in brain endothelial cells (Beuckmann et al., 1995).

### 3.2 Comparison with porcine models in the literature

Table 2 compares the basic characteristics achieved in this model, with three others from the literature: Franke et al. (2000), Zhang et al. (2006) and Smith et al. (2007). The selection of which method to use may be influenced by many factors, including the culture expertise of a group, models historically used, and the intended applications. The properties of the models are in many respects quite similar, adding to evidence that whatever the details of the preparative method, the confluent porcine brain endothelial model shows generally comparable behaviour, so that results from different studies can to some extent be pooled to form a growing database of information. Several methods have been described, but intra-batch and batch-to-batch variation was still a problem with many of them (Franke et al., 2000; Zhang et al., 2006). There was some variability in the effects of adding serum, reported to either increase or decrease permeability (Nitz et al., 2003).

The strengths of the present model are that it is relatively simple, involving fewer preparative steps: simply dissect out grey matter, homogenise, filter and digest to obtain brain microvessels. There are no complicated gradient separations. The model reliably gives tight brain endothelial cell monolayers without astrocyte influence. This can be attributed to the simple but strict preparative methodology, the removal of contaminating cells such as pericytes through the use of puromycin in the first three days of culture and the use of the differentiation medium. The high TEER itself has many advantages. It indicates good functional tight junctions, known to help in development of good apical-basal polarity in the cells (‘fence’ function of tight junctions, Abbott et al., 2006) and hence in preserving many important polarised features of the physiological BBB phenotype. Moreover, by restricting paracellular permeation, the effective tight junctions also give better resolution and discrimination for carrier-mediated transport (‘gate’ function of tight junctions, Abbott et al., 2006). Use of the different filter meshes to separate the two microvessel fractions gives the option of using the 60s for investigations such as drug permeability assays where a tight monolayer is essential and the 150s for uptake and efflux studies when maximum tightness is not required. Finally, a QC test was adopted to check the reliability and repeatability of different cultures.

### 3.3 Effects of elevation of cAMP and addition of hydrocortisone

Several studies have shown that increasing intracellular cAMP levels (Deli et al., 2005; Gaillard et al., 2001; Hurst and Clark, 1998; Ishizaki et al., 2003; Perrière et al., 2007; Rubin et al., 1991) and addition of physiological levels of hydrocortisone (Förster et al., 2005; Hoheisel et al., 1998; Perrière et al., 2007) to brain endothelial cell cultures can increase the barrier function of tight junction proteins. Ishizaki et al. (2003) showed that cAMP increased claudin-5 gene expression via a protein kinase A (PKA)-independent pathway, but increased TEER via both PKA-dependent and -independent pathways in PBECs. By contrast, cAMP decreased TEER in a rat lung endothelial cell line expressing doxycycline-inducible wild-type claudin-5 (Soma et al., 2004). The authors suggested that cAMP could be responsible for increasing the barrier function of other tight junction proteins, but not claudin-5. However, this study was on a lung endothelial cell line and may not be comparable to claudin-5 function in brain endothelial cells.

Hydrocortisone is a glucocorticoid and like cAMP can increase TEER of brain endothelial cells at physiological concentrations (70–550 nM). Studies by Förster et al. (2005) have shown that treatment of cEND (an immortalised mouse brain endothelial cell line) with hydrocortisone led to an increase in TEER by threefold and up-regulation of occludin. A three-fold increase in TEER and over twofold increase in expression of occludin and claudin-5 was also observed in hCMEC/D3, immortalised human brain microvascular endothelial cells, treated with hydrocortisone (Förster et al., 2008). The culture medium for our *in vitro* PBEC model is supplemented with 550 nM hydrocortisone and the cells are treated with 250 μM pCPT-cAMP and 17.5 μM RO-20-1724 to increase intracellular cAMP levels. Therefore, high TEER, ALP activity and clear expression of tight junction proteins in our model could also be attributed to these treatments. The differentiation medium is replaced by a simpler medium (‘donor buffer’) containing DMEM+25 mM HEPES and 0.1% bovine serum albumin without the differentiating factors for permeability assays. These assays are of short duration (30 min) and therefore the lack of differentiation factors does not significantly affect the resolution of drug permeation across the PBEC monolayer. In a different PBEC model, Nitz et al. (2003) reported that serum-derived factors destabilised tight junction protein strands after tight junctions were established. The present model also avoids using serum after tight junctions are stabilised. Monocultured PBECs in this model are flat cells with a broadly elongate cobblestone-shaped morphology. The more cobblestone morphology could be an effect of hydrocortisone treatment as suggested by Förster et al. (2005) or reflect the absence in monoculture of soluble factors released by astrocytes that influence the *in vivo* morphology of the BBB.

### 3.4 Inductive influence of astrocytes

Brain capillary endothelial cells *in vivo* are closely associated with several cell types within the neurovascular unit (Abbott et al., 2006) including pericytes (Daneman et al., 2010; Lai and Kuo, 2005), astrocytes (Abbott, 2002; Abbott et al., 2006), perivascular macrophages (Zenker et al., 2003) and neurons (Schiera, 2003). Numerous studies have shown that each of these cell types can induce aspects of BBB phenotype when co-cultured with brain endothelial cells, with induction by astrocytes being the most fully documented, and astrocytes the most common cell type used to induce BBB features in co-cultured *in vitro* BBB models (Abbott et al., 2006). However, it was not clear which cell type exerts the strongest influence *in vivo*, or how BBB induction occurs during CNS development.

Recent studies using a combination of genetically engineered animals and cell culture have provided a clearer developmental sequence, showing initial BBB induction by neural progenitor cells at the time of vascular ingrowth into the neural tube (angiogenesis), followed by progressive maturation of the BBB phenotype involving influences first from pericytes and later from astrocytes (Armulik et al., 2010; Daneman et al., 2010; Paolinelli et al., 2011; Thanabalasundaram et al., 2011). Pericytes cause upregulation of key BBB features such as tight junction protein expression and organisation, and expression of nutrient transporters such as Glut-1/SLC2A1, while downregulating ‘default’ features characteristic of peripheral endothelial cells such as leucocyte adhesion molecule expression and vesicle trafficking (Daneman et al., 2010). Astrocytes, which mature later, then refine the BBB phenotype further, especially by upregulation of efflux transporters (Daneman et al., 2010); they also appear able to induce the expression of a greater range of BBB-specific genes than pericytes (Nag, 2011). Both pericytes and astrocytes are important in maintaining the BBB phenotype in the adult (Bell et al., 2010; Nag, 2011).

When microvessels are isolated from adult brain, as typically used for *in vitro* BBB models, the endothelium will have a fully functional BBB phenotype. There appear to be species differences in the rate at which this is lost in culture, relatively rapidly in rat and bovine brain endothelial cells, more slowly in PBECs, as shown by the good preservation of tight junctions, high TEER and functional efflux transporters in monocultured PBEC models. Many studies show more effective tight junctions and higher TEER of the tightest *in vitro* models in the presence of astrocytic influence (co-culture or conditioned medium) as demonstrated in bovine brain endothelial cell models (Dehouck et al., 1992; Rubin et al., 1991) and many PBEC models (Fischer et al., 2000; Kido et al., 2002; Smith et al., 2007; Zhang et al., 2006). Earlier studies have also shown that ALP activity is reduced in monocultures of porcine brain endothelial cells, and co-culturing with astrocytes is required for re-inducing the ALP activity (Meyer et al., 1990, 1991). However, the model described here does not require inductive influences from astrocytes to maintain a high TEER or to show high ALP activity. For certain more complex features such as receptor-mediated transcytosis (RMT) (Candela et al., 2008; Demeule et al., 2002), co-culture with astrocytes appears necessary to sustain a sufficiently differentiated phenotype for mechanistic and screening studies (Cecchelli et al., 2007; Skinner et al., 2009). While ‘triculture’ models that include pericytes (Nakagawa et al., 2009) may show some useful additional properties (Al Ahmad et al., 2011; Ramsauer et al., 2002), endothelial-astrocyte models can show a BBB phenotype close enough to the *in vivo* situation to make more practical systems for mechanistic studies and permeability assays.

### 3.5 Extent of BBB dedifferentiation in culture

Previous studies have reported that primary brain endothelial cells tend to lose their BBB phenotype when passaged (Franke et al., 2000; Igarashi et al., 1999; Omidi et al., 2003; Rubin et al., 1991). Hence changes in phenotype must be investigated not only with respect to changes between *in vivo* and primary cultures, but also between primary and passaged cultures, as serial passaging leads to a further loss of phenotype. Another complication when using *in vitro* BBB models is the variability between cultures. Therefore, real-time PCR assays were performed to test variability and differentiation of PBECs when passaged once (primary to P.1) using three genes of interest, BCRP, occludin and claudin-5. The results demonstrated that PBECs do not dedifferentiate significantly when passaged once, as the relative mRNA expression levels of BCRP, occludin and claudin-5 were not significantly different between primary and P.1 PBECs (fold difference ratio <2.0). The immunocytochemical images from freshly isolated porcine brain microvessels showed clear expression of occludin and claudin-5. Clear edge staining was also observed in P.1 PBECs, confirming the maintenance of BBB features after passaging. The loss of *in vivo* phenotype reported for many *in vitro* BBB models appears to be mainly due to the removal of endothelial cells from their natural environment. However, the changes can be counteracted to some degree using several inductive factors and co-cultures as discussed.

### 3.6 PBECs as a drug screening tool

Recently developed primary cultured *in vitro* BBB models offer advantages as assay systems since they express more features of the *in vivo* BBB (including membrane lipid and protein composition, expression of uptake and efflux transporters and drug metabolising enzymes) than Caco-2 (from human colon carcinoma) or MDCK (from canine kidney epithelium) cell lines, which are commonly used in the pharmaceutical industry. Until around year 2000 the *in vitro* BBB model showing the best correlation with *in vivo* BBB permeability was the system using bovine brain endothelial cells co-cultured on filters above rat astrocytes (Cecchelli et al., 1999), but over the last decade several groups have reported successful use of porcine brain endothelial cells as useful tools for drug screening (Franke et al., 1999, 2000; Smith et al., 2007; Zhang et al., 2006). Our results demonstrate that the PBEC model described here has the potential to be useful as a permeability screen to investigate BBB permeation of drugs of interest with a range of chemistries, including those that are substrates for transporters, whether or not the particular transporters involved have been identified. With inclusion of sufficient passively permeating reference compounds, substrates for transporters can be identified as outliers, for further mechanistic study.

If required and desirable, porcine brain endothelial cell production could be scaled up for high/medium-throughput screening. However, it is possible to limit the numbers of compounds that need to be tested on living BBB models using better *in silico* (computer-based) screens. Thus a serial and parallel screening process can be used to bring the numbers to manageable level (*e.g.* 200 *cf.* >100,000) for testing on an *in vitro* BBB model (Abbott, 2004).

In conclusion, results confirm that this optimised *in vitro* porcine BBB model is relatively simple to prepare, reliable and repeatable compared to most other static BBB models, and gives high TEER without the need for astrocyte co-culture. The quality, simplicity and robustness of the porcine BBB model make it an attractive model for industry to use in CNS drug discovery programmes and also for a variety of basic scientific projects. Because the method generates PBECs with high TEER, it is likely to show good apical: basal differentiation for other important BBB features, including receptors, transporters, enzymes and ion channels. It is therefore likely to be suitable for a range of experimental projects where such apical: basal polarity is critical, especially for vectorial transport, and differences in cell signalling to and from the endothelium that involve the apical and basal membranes. The model is currently being optimised further to improve the dynamic range for permeability studies, and is being used in applications to examine several other aspects of BBB function including transcytosis of large molecules and constructs, and drug efflux transporters.

## 4 Experimental procedure

### 4.1 Materials

Dulbecco's Modified Eagle's Medium (DMEM) without phenol red, α-MEM with Glutamax-1 and Hams F-10 with Glutamax-1 were from Invitrogen Corporation (Paisley, UK), foetal calf serum (FCS), penicillin/streptomycin, Ca^2+^/Mg^2+^-free Hanks balanced salt solution (HBSS), Ca^2+^/Mg^2+^-free HBSS without phenol red, trypsin-EDTA, DMEM (for cell culture), L-15 medium, M199 medium, fibronectin, glutamine, heparin, hydrocortisone, puromycin, verapamil, HEPES, pCPT-cAMP, trypsin-EDTA, Ca^2+^/Mg^2+^-free Hanks balanced salt solution (HBSS), Ca^2+^/Mg^2+^-free HBSS without phenol red, Geneticin, basic fibroblast growth factor (bFGF), poly-l-lysine, carbodiimide, paraformaldehyde, Triton X-100, normal goat serum (NGS), Hoescht 33258 nuclear stain, Sigma Fast *p*-nitrophenyl phosphate (pNPP) tablets and other standard laboratory reagents of analytical grade were from Sigma-Aldrich Chemical Co. (Dorset, UK). 4-(3-Butoxy-4-methoxybenzyl)-2-imidazolidinone (RO-20-1724) was from Calbiochem/Merck. Collagenase, dispase and DNase I were from Lorne Laboratories Ltd. (Reading, UK). Minimum Essential Medium (MEM) was from MP Biomedicals (UK) and Bovine Plasma Derived Serum (BPDS) was from First Link (Birmingham, UK). Nylon meshes were obtained from Plastok associates (Wirrel, UK) and Corning Transwell-clear inserts (12 mm diameter, 1.13 cm^2^ growth area, 0.4 μm pore size, 4×10^6^ pores/cm^2^) were obtained from Fisher Scientific (UK). All other tissue culture materials were obtained from Invitrogen (Paisley, UK) unless stated otherwise. [^14^C]sucrose, [^14^C]caffeine (50 mCi/mmol) and [^3^H]propranolol (30 Ci/mmol) were purchased from GE Healthcare, UK. [^3^H]colchicine (76.5 Ci/mmol), [^3^H]l-glutamic acid (49.9 Ci/mmol), [^3^H]diazepam (76 Ci/mmol), [^3^H]digoxin (37 Ci/mmol), [^3^H]vinblastine (10.9 Ci/mmol), [^3^H]naloxone (63 Ci/mmol) and OptiPhase HiSafe 2 scintillation liquid were purchased from PerkinElmer Life & Analytical Sciences (Buckinghamshire, UK). [^3^H]l-leucine (159 Ci/mmol) was purchased from Sigma-Aldrich Ltd (Dorset, UK). The bicinchoninic acid (BCA) protein assay kit was from Pierce Biotechnology. Rabbit anti-occludin and rabbit anti-claudin-5 were from Zymed laboratories and Alexa Fluor 594 labelled goat anti-rabbit secondary antibody was from Molecular Probes. EZ1 RNA cell mini kit and QuantiTect reverse transcription kit were from QIAGEN. All primers were from Sigma Genosys. TaqMan probes and the 2×TaqMan Universal PCR Master Mix (product number – 4304437) were from Applied Biosystems.

### 4.2 Isolation of porcine brain microvessel endothelial cells

Pig brains were obtained fresh from the abattoir and transported on ice in L15 medium with added penicillin (100 U/ml) and streptomycin (100 μg/ml). Brains were washed in phosphate-buffered saline (PBS) (with Ca^++^/Mg^++^) and meninges were thoroughly peeled off and discarded. White matter was carefully removed. The grey matter was collected in HEPES-buffered MEM containing 10% foetal calf serum (MEM-H 10% FCS), forced through a 50 ml syringe to produce a slurry, and mixed with an equal volume of MEM-H 10%. Tissue was gently homogenised in a glass Wheaton Dounce tissue grinder (Jencons Scientific Ltd., Leighton Buzzard, UK) (89–127 μm clearance, 15 strokes; 25–76 μm clearance 15 strokes) and sequentially filtered, first through 150 μm nylon mesh, then through 60 μm nylon mesh. Microvessel fragments trapped on the 150 and 60 μm meshes were kept separate and digested at 37 °C for 1 h in medium M199 containing 10% FCS, 223 U/mg collagenase, 211 U/mg trypsin and 2108 U/mg DNase with continuous agitation. Microvessels were washed off the meshes with the enzyme mixture, centrifuged for 5 min at 240*g* at 4 °C to remove enzyme, then resuspended in MEM-H 10% FCS and centrifuged again; the resulting vessel fractions were kept separate as ‘150s’ and ‘60s’, the latter giving higher TEER. The ‘60s’ were used for all experiments described here. Digested fragments were resuspended in 10% DMSO in foetal calf serum, brought slowly to −80 °C and stored in liquid nitrogen. Six pig brains gave 12 1 ml aliquots of ‘60s’.

### 4.3 Culture of capillary fragments

Capillary fragments were thawed and resuspended in plating medium consisting of DMEM with 10% BPDS with 100 U/ml penicillin, 100 μg/ml streptomycin, 2 mM glutamine, 125 μg/ml heparin, with 4 μg/ml puromycin to kill contaminating cells, especially pericytes (Perrière et al., 2005). One aliquot was plated into two T75 flasks coated with lab-prepared rat tail collagen (Strom and Michalopoulos, 1982) and 7.5 μg/ml fibronectin, and grown to 70–80% confluence. Cells were detached by brief trypsinisation (500 BAEE units trypsin and 0.47 mM EDTA.4Na in HBSS without Ca^2+^ or Mg^2+^), then centrifuged at 360*g* for 5 min. The pellet of these first passage (P1) cells was resuspended in plating medium containing DMEM, 10% BPDS, 100 U/ml penicillin, 100 μg/ml streptomycin, 2 mM glutamine and 125 μg/ml heparin. Cells were seeded onto collagen/fibronectin coated Transwell-Clear inserts at a density of 1×10^5^ cells/cm^2^ or at 1×10^4^ cells/well in 96-well plates for functional studies and grown for 2–3 day until confluent. The medium was changed to serum-free medium supplemented with 550 nM hydrocortisone (Hoheisel et al., 1998) and the cells were treated with 250 μM pCPT-cAMP and 17.5 μM RO-20-1724 (Rubin et al., 1991); these supplements helped to improve differentiation of BBB properties, especially tight junction maturation (Förster et al., 2005). PBECs were used in experiments 24 h after this medium change. The quality of the model in terms of cell growth was assessed according to the time the cultures took to become confluent. The quality control (QC) benchmark was three days to become ∼70–80% confluent after first plating and another three days to become ∼100% confluent after passaging. All the cultures assessed passed this QC test (Table 1) and were suitable for use in subsequent experiments.

### 4.4 RBE4 immortalised rat brain endothelial cell culture

RBE4 cells (Roux et al., 1994) were kindly provided by Dr. P.O. Couraud and Dr. F. Roux (Inserm, Paris). RBE4 cells were maintained in α-MEM with Glutamax-1 (45%), Hams F-10 with Glutamax-1 (45%) containing 10% FCS, Geneticin (300 μg/ml) and basic fibroblast growth factor (bFGF, 1 ng/ml). Cells were grown in collagen-coated T25 flasks and were maintained in 5% CO_2_ humidified atmosphere at 37 °C. The cells were passaged every three day and the culture medium replaced every 2–3 days. RBE4 were seeded at 1.0×10^4^ cells/200 μl growth medium per well in 96-well plates and grown to confluence. Experiments were performed when cells were confluent, typically within three days of seeding.

### 4.5 Tissue print method for porcine brain microvessels

A tissue print method was used to attach porcine brain microvessels to glass slides by modifying a technique for attaching rat retinal microvessels to glass coverslips (Sakagami et al., 1999). A small piece of fresh porcine brain was placed in a Petri dish containing 2 ml medium. Using forceps and a scalpel, the brain matter was cut into 1–2 mm^3^ pieces, and then a cut piece was placed on a poly-l-lysine -coated glass slide. A second glass slide, also coated with poly-l-lysine was placed over the piece of brain tissue. Forceps touching the upper side provided gentle downward pressure that sandwiched the piece of brain tissue between the two glass slides. During this tissue print step, microvessels adhere to the glass slides. After 1 min, the upper glass slide was carefully removed. The two slides were placed in a Coplin jar filled with PBS to wash off excess tissue. The tissue prints were further processed for immunocytochemistry.

### 4.6 Immunochemistry

P.1 PBECs were grown on glass coverslips coated with collagen/carbodiimide to aid cell adhesion (Nobles and Abbott, 1994). P.1 PBECs or porcine brain microvessels were washed with PBS, fixed with 3% paraformaldehyde for 45 min and then permeabilised in 0.1% Triton X-100. To block non-specific binding, cells/microvessels were treated for 60 min with normal goat serum and incubated overnight at 4 °C with primary antibodies (rabbit anti-occludin and rabbit anti-claudin-5) diluted 1:100 in PBS containing 3% NGS. Cells/microvessels were subsequently rinsed with PBS for 60 min and incubated for 2 h at room temperature with secondary Alexa Fluor 594 labelled goat anti-rabbit antibody and Hoescht 33258 nuclear stain. Cells/microvessels were washed again for 60 min with PBS before mounting on glass slides using Mowiol. Samples were visualised by fluorescence microscopy (Axioskop; Carl Zeiss Ltd.) and images were captured by Axiovision software (Carl Zeiss Ltd.).

### 4.7 Transendothelial electrical resistance measurements

TEER across PBEC monolayers on Transwells was determined using an EVOM resistance system (World Precision Instruments, Hertfordshire, UK) with Endohm electrode chamber. The measured resistance of cells grown on Transwell filter inserts was corrected for resistance across an empty collagen/fibronectin-coated Transwell insert, and multiplied by surface area, to give TEER in ohms×cm^2^ (Ω cm^2^).

### 4.8 Apparent permeability to [^14^C]sucrose

Permeability assays were performed on cell monolayers with TEER >500 Ω cm^2^. Culture medium was aspirated and the inserts transferred to 12-well plates containing 1.5 ml/well donor buffer (DMEM without phenol red, 25 mM HEPES and 0.1% bovine serum albumin) and placed in an orbital shaker at 37 °C. Donor buffer (0.5 ml) containing [^14^C]sucrose (0.15 μCi/ml, specific activity 643 mCi/mmol) was added to the inserts sequentially at 10-s intervals. At *t*=5 min, the inserts were transferred to the next well containing donor buffer. This procedure was repeated for all inserts at *t*=15 min and *t*=30 min. At the end of the experiment, samples were taken from each insert and well to scintillation vials. OptiPhase HiSafe 2 scintillation liquid was added to each vial and radioactivity was counted using a Canberra Packard Tricarb 1900 TR Liquid Scintillation Analyser.

Cleared volume was calculated using the following equation and plotted as a function of time:

(1) $$\mathrm{Cleared}\;\mathrm{volume}\left(\mu l\right)=M_{R}\left(\mathrm{dpm}\right)/C_{D}\left(\mathrm{dpm}/\mu l\right)$$

where is the *M*_R_=amount of radio-labelled compound in the receiver compartment, dpm=disintegrations per minute, *C*_D_=concentration of the compound in the donor compartment.

All dpm values were corrected for background dpm. The slope of the clearance curve was obtained by linear regression and represents the *PS* (*i.e.* permeability × surface area) product. Apparent permeability (*P*_app_, cm/s) was calculated using the following equation:

(2) $$P_{\mathrm{app}}\left(\mathrm{cm}/s\right)=PS\;\mathrm{product}\left({\mathrm{cm}}^{3}/s\right)/\mathrm{surface}\;\mathrm{area}\;\mathrm{of}\;\mathrm{the}\;\mathrm{insert}\;\left({\mathrm{cm}}^{2}\right)$$

### 4.9 P-glycoprotein function

*Colchicine uptake assay*: P-gp function was measured using uptake of [^3^H]colchicine (P-gp substrate) on cells grown in 24-well plates (Begley et al., 1996). Uptake medium contained HBSS without phenol red, 10 mM HEPES, [^14^C]sucrose (0.045 μCi/ml, specific activity 0.2 mCi/mol) to correct for non-specific binding, and [^3^H]colchicine (1.0 μCi/ml, specific activity 76.5 Ci/mmol). Briefly, culture medium was aspirated off control wells and 1ml uptake medium per well was added at 10-s intervals to each well. This procedure was repeated for the test wells with 50 μM verapamil (P-gp inhibitor) in the uptake medium. Cells were incubated for 30 min at 37 °C, then uptake medium was aspirated and cells were washed three times with PBS. Cells were lysed with 1% Triton X-100 for 1 h and 300 μl aliquots taken for counting radioactivity. Fifty-microlitre aliquots from each uptake medium (±verapamil) were taken as standards. OptiPhase HiSafe 2 scintillation liquid was added to each vial and radioactivity counted using a Canberra Packard Tricarb 1900 TR Liquid Scintillation Analyser. Protein concentration of a 100 μl aliquot from each well was determined using the BCA protein assay kit. The cellular accumulation of [^3^H]colchicine was calculated as the distribution volume (*V*_d_, μl/mg protein) derived from the ratio of cell radioactivity/mg (dpm/mg) protein over the radioactivity per μl uptake medium after correcting for background radioactivity (Eq. (3)). *V*_d_ for [^3^H]colchicine was corrected for non-specific binding by subtracting the *V*_d_ for [^14^C]sucrose, as non-permeant extracellular marker.

(3) $$V_{d}\left(\mu l\right)=\mathrm{dpm}\;\mathrm{in}\;\mathrm{cells}/\left[\mathrm{dpm}\;\mathrm{in}\;\mathrm{aliquot}\;\mathrm{of}\;\mathrm{uptake}\;\mathrm{medium}/\mathrm{volume}\;\mathrm{of}\;\mathrm{aliquot}\;\left(\mu l\right)\right]$$

All dpm values were corrected for background dpm. *V*_d_ was then normalised for the cell protein concentration (mg) to give units of μl/mg protein.

### 4.10 Alkaline phosphatase (EC 3.1.3.1) assay

P.1 PBECs or RBE4 cells were grown in 96-well plates at 1.0×10^4^ cells/200 μl growth medium per well. Cells were washed three times with PBS, and cell membranes disrupted by freezing at −80 °C for 20 min. Alkaline phosphatase (ALP) assay was performed using Sigma Fast *p*-nitrophenyl phosphate tablets. Two hundred microlitres of pNPP was added to each well and incubated in the dark for 60 min at room temperature. Absorbance at 405 nm was read in a Labsystems Multiskan Ascent plate reader and protein concentration determined using the BCA protein assay kit. ALP activity levels are reported as absorbance per milligram protein.

### 4.11 RNA extraction and cDNA analysis

Two vials each of PBECs from two different batches (batch 1 and 2) of PBEC were used to obtain primary and P.1 PBECs. RNA was extracted from three primary and P.1 cultures from each vial (24 samples) using the EZ1 RNA cell mini kit. Twelve microlitres of RNA (∼300–450 ng) from each sample was reverse transcribed using the QuantiTect reverse transcription kit to generate cDNA. RNA and cDNA were analysed (260/280 ratio: RNA∼2.0; cDNA∼1.8) and quantified using the NanoDrop^®^ ND-1000 spectrophotometer (NanoDrop Technologies, USA).

### 4.12 Primers and probes

Primers and TaqMan^®^ probes for porcine glyceraldehyde-3-phosphate dehydrogenase (GAPDH, reference gene), occludin, claudin-5 and BCRP were designed using Primer Express^®^ software from Applied Biosystems. The total gene specificity of the nucleotide sequences chosen for the primers and probes was confirmed using nucleotide-nucleotide BLAST searches (GenBank database sequences) (National Center for Biotechnology Information 2006). The nucleotide sequences of the oligonucleotide hybridisation primers and probes for TaqMan analysis are shown in Table 3.

### 4.13 TaqMan real-time quantitative PCR analysis

TaqMan real-time polymerase chain reaction (PCR) assays were performed using the AB 7900HT Real-Time PCR System with a 384-well configuration. The TaqMan probes used in this study were dual-labelled with a 5′ end 6-FAM (a high-energy ‘Reporter’ dye) and a 3′ end TAMRA (a low-energy ‘Quencher’ dye). The optimum primer and probe concentrations were determined by running replicate standard samples at different primer and probe concentrations. The PCR reaction mixture contained 2 μl of cDNA sample (10 ng) and 2×TaqMan Universal PCR Master Mix with 900 nM primers and 250 nM TaqMan probe in a total volume of 20 μl. After an initial step of AmpErase^®^ uracil-*N*-glycosylase activation (to prevent the re-amplification of carryover-PCR products) at 50 °C for 2 min and denaturation at 95 °C for 10 min, the cDNA products were amplified with 40 PCR cycles, consisting of a denaturation step at 95 °C for 15 s and an extension step at 60 °C for 1 min. Each cDNA sample was run in technical triplicate using gene-specific primers. Therefore each gene set included 36 target and 36 reference cDNA samples. Each gene set also contained a 5-point standard curve for the reference and target genes, a no-template control, an extraction negative and a reverse transcriptase (RT) negative as controls.

### 4.14 Gene expression analysis

mRNA expression was analysed independently by one-way analysis of variance (ANOVA) using Satistica 6 software (StatSoft Inc., USA). Data analysis was carried out using Sequence Detection Systems software (Applied Biosystems). For quantification of gene expression changes, the ‘relative standard curve method’ was used to calculate relative fold changes normalised against the GAPDH gene (endogenous control) using Eqs. (5) and (6). No significant differences were observed between vials within a batch (for all genes) and the vials behaved consistently across the two batches tested (batch 1 and 2). Therefore, data from all vials were pooled to increase the level of replication for each condition. Fold differences were calculated using Eq. (7) to compare batch-to-batch differences and primary *vs.* P.1 gene expression levels. A 2-fold difference is considered significant.

(5) $$\mathrm{Normalised}\;\mathrm{target}\left(\mathrm{test}\right),\;{\mathrm{NT}}_{T}=\mathrm{Target}/\mathrm{endogenous}\;\mathrm{control}$$

(6) $$\mathrm{Normalised}\;\mathrm{target}\left(\mathrm{calibrator}\right),\;{\mathrm{NT}}_{C}=\mathrm{Target}/\mathrm{endogenous}\;\mathrm{control}$$

(7) $$\mathrm{Fold}\;\mathrm{difference}\;\mathrm{in}\;\mathrm{target}={\mathrm{NT}}_{T}/{\mathrm{NT}}_{C}$$

### 4.15 Permeability screening

Permeability assays (apical to basal direction) were performed on 10 radio-labelled compounds covering passive permeation ([^3^H]diazepam, [^3^H]naloxone, [^3^H]propranolol, [^14^C]sucrose), uptake ([^14^C]caffeine, [^3^H]L-glutamic acid (as Na glutamate in saline), [^3^H]L-leucine) and efflux ([^3^H]colchicine, [^3^H]digoxin, [^3^H]vinblastine) transporters, as described for [^14^C]sucrose in Section 4.8. The apparent permeability *P*_app_ was calculated according to Eq. (2) and plotted against the calculated Log *P*_octanol_ as a measure of lipophilicity of the compound. Log *P*_octanol_ estimation was obtained from http://www.syrres.com/eSc/est_kowdemo.htm.

### 4.16 Statistical analysis

Data were expressed as mean±standard error of the mean (SEM) and analysed and presented using Microsoft Excel or GraphPad Prism (version 4.0). Groups of two were analysed using Student's *t*-test, groups of three or more were analysed using one-way analysis of variance (ANOVA) with a Dunnett's *post-hoc* test. Values were considered to be significantly different when the probability that differences were not due to chance alone was less than 5% (*p* <0.05).

## Figures and Tables

**Table 1:** Quality control panel used to assess porcine brain endothelial cells.

**Feature**	**Quality control benchmark**
Morphology/confluence	Pure endothelial cells/ready for experiments within seven days of thawing
Differentiation	Expression of major BBB transporters and enzymes
Permeability	TEER>500 Ω cm^2^ and *P*_app_ to [^14^C]sucrose <8×10^−6^ cm/s

**Table 2:** Comparison of methods and barrier characteristics of selected porcine blood–brain barrier models.

**Porcine BBB model**	**Patabendige et al. (this paper)**	**Franke et al. (2000**)	Zhang et al. (2006)	**Smith et al. (2007**)
Based on	Rubin et al. (1991)	Bowman et al. (1983)	Miller et al. (1992)	Franke et al. (2000)
Meninges	Remove including into sulci	Flame, remove with surface vessels	Remove, with surface vessels	–
Grey matter (GM)/white matter (WM)	Cut/pinch off and discard as much WM as possible	GM+WM, minced	Collect GM by aspiration	Remove WM and accessory organs
Homogeniser	Y	N, mince with blades	Y by sequential filtration	N, mechanically mill
Filtration mesh size	Fine: 150 μm, 60 μm	180 μm	Coarse: 1000 μm, 710 μm	–
Enzyme digest 1	Collagenase, trypsin, DNase I 1 h, 37 °C	Dispase II 2 h, 37 °C	12.5% dispase 3 h. Centrifuge 1570*g,* 10 min, 37 °C	1% dispase 2 h
Gradient separation 1	–	Dextran, 6800 g, 10 min, 4 °C	13% dextran, 9170 g, 10	30% Percoll, 6800 g
Enzyme digest 2	–	Collagenase/dispase 0.1% 1 h 37 °C	Collagenase/dispase 0.52% 3.5 h, 37 °C	Collagenase/dispase 0.2% 1 h
Gradient separation 2	–	Percoll 1250 g	Percoll 1700 g 10 min	Percoll 1250 g
Yield/cells per brain	10×10^6^	450 cm^2^ flask area	10×10^6^	25×10^6^
Medium	DMEM+10% BPDS	M199+10% ox serum	MEM: F12 +10% horse serum.	M199+10% horse serum
Subculture	Y, day 3 to purify	Y, day 3 to purify	N	N
Endothelial cell purification method	4 mg/ml Puromycin	–	–	–
Switch (differentiation) medium	DMEM+550 nM HC+CPT-cAMP+RO-20-1724 24 h. No BPDS	1:1 DMEM: F12, Serum-free or +1% ox serum, +2.5% BSA. 550 nM HC 24 h	–	M199+550 nM HC 24 h. No FCS
Astrocytes	N	N	± ACM abluminal	+C6 glioma cells or C6 CM
Filter inserts for cell growth	12 mm diam (area 1.13 cm^2^)	24 mm diam (area 4.5 cm^2^)	24 mm diam (area 4.5 cm^2^)	6.5 mm and 24 mm diam (area 0.33 cm^2^ and 4.5 cm^2^)
Filter coating	Lab-made rat tail collagen/fibronectin	Rat tail collagen (Bornstein), dried	Rat tail collagen/fibronectin	Rat tail collagen type I
Seeding density on inserts	1×10^5^ cells/cm^2^	3×10^4^ cells/cm^2^	7.5×10^4^ cells/cm^2^	1×10^5^ cells/cm^2^
Days to confluence on filters	3+2/3+1 d (switch medium )	3+3+1 d (switch medium)	5–6 d	6+1d (switch medium)
Maximum tightness	6–8 d	7–8 d	5–9 d	7 d
TEER	Endohm. ∼789±18 Ω cm^2^ (with puromycin)	Endohm. 400±100 to 700±100 Ω cm^2^.	Endohm. Use >300 Ω cm^2^. With rat ACM: TEER increase 10–25% 6–9 d.	80 Ω cm^2^ (w/o C6); 409 Ω cm^2^ (C6 CM); 834 Ω cm^2^ (C6 co)
P_app_ sucrose	∼6×10^−6^ cm/s	4.5×10^−6^ cm/s(+Serum), 1×10^−6^ cm/s(−Serum).	80×10^−6^ cm/s	12.1×10^−6^ cm/s(w/o C6); 8.8×10^−6^ cm/s(C6 CM); 1.6×10^−6^ cm/s(C6 co)
Freezing method for long-term storage	FCS+10% DMSO	–	MEM:F12 +10% DMSO, 20% horse serum, heparin, gentamycin, amphotericin	M199+20% horse serum+10% DMSO

Abbreviations: yes, Y; no, N; Dulbecco's modified Eagle's medium, DMEM; medium 199, M199; minimum essential medium, MEM; Ham's F12 medium, F12; hydrocortisone, HC; bovine plasma-derived serum, BPDS; bovine serum albumin, BSA; foetal calf serum, FCS; dimethyl sulfoxide, DMSO; astrocyte-conditioned medium, ACM; conditioned medium, CM; without, w/o; transendothelial electrical resistance, TEER.

**Table 3:** Primers and probes used for real-time RT-PCR assays.

**Gene**	**Accession number**	**Forward primer**	**Reverse primer**	**TaqMan probe**	**Product size (base pairs)**
BCRP	AJ420927	GAGCTTATTACTGACCCGTCTATCTTG	GCATTTGCTGTGCTGGAGTCT	CCTGGATGAGCCCACGACTGGC	73
Claudin-5	AJ318103	CTCTGCTGGTTCGCCAACA	CAGCTCGTACTTCTGCGACATG	TCCGCGAGTTCTACGACCCGACTGT	74
Occludin	U79554	GAGGAAGACTGGATCAGGGAATATC	GGCCACTGTCAAAATTTCTCTTG	CCCATCACTTCAGATCAACAAAGGCAACTC	81
GAPDH	U48832	ATTCCACCCACGGCAAGTT	ATGGCCTTTCCATTGATGACA	CACGGCACAGTCAAGGCGGAGA	72
